# Fecal carriage and associated factors of extended-spectrum beta-lactamase-producing Enterobacteriaceae in children under five years old with diarrhea at a tertiary hospital in Tanzania

**DOI:** 10.1371/journal.pone.0334051

**Published:** 2025-10-10

**Authors:** Emily M. Jackson, Doreen Kamori, Ambele M. Mwandigha, Vulstan J. Shedura, Upendo Kibwana, Mariam Mirambo, Agricola Joachim, Joel Manyahi, Mtebe V. Majigo

**Affiliations:** 1 Department of Microbiology and Immunology, Muhimbili University of Health and Allied Sciences, Dar es Salaam, Tanzania; 2 Department of Clinical Research, Training and Consultancy, Southern Zone Referral Hospital, Mtwara, Tanzania; 3 Department of Microbiology and Immunology, Catholic University of Health and Allied Sciences, Mwanza, Tanzania; Ahvaz Jondishapour University of Medical Sciences Faculty of Medicine, IRAN, ISLAMIC REPUBLIC OF

## Abstract

**Background:**

The prevalence of extended-spectrum beta-lactamase-producing Enterobacteriales (ESBL-PE) is high in resource-limited settings. Carriage in young children has been linked to increased morbidity and mortality. However, there is limited information on the prevalence of ESBL-PE among children under five in our settings. This study aimed to determine the fecal carriage of ESBL-PE in children under five years old suffering from diarrhea at a tertiary hospital in Tanzania.

**Methodology:**

This cross-sectional study was conducted over a three-month period at Muhimbili National Hospital in Dar es Salaam, Tanzania. Participants’ socio-demographic and clinical information were collected using a structured questionnaire and the child’s clinic card. Stool samples were collected and screened for the presence of ESBL-PE using MacConkey agar supplemented with ceftazidime. Confirmation of ESBL-PE was achieved through a double disk synergy test. Logistic regression analysis was employed to identify factors independently associated with ESBL-PE carriage. A *p*-value of less than 0.05 was considered statistically significant.

**Results:**

We enrolled 181 children with a median age of 29 months (interquartile range: 20–37). The female-to-male ratio was 1:1. Among all participants, 54.1% (98/181) were malnourished, and the parents of 54.1% (98/181) had low levels of education. The overall fecal carriage rate of ESBL-PE was 65.7% (119/181). A total of 120 ESBL-PE isolates were confirmed. *E. coli* was the predominant ESBL-PE, contributing to (74.2%, 89/120), followed by *K. pneumoniae* (21/120, 17.5%), *K. oxytoca (8/120, 6.7%),* and Citrobacter spp. (2/120, 1.7%). Malnourished children had a higher carriage rate of ESBL-PE (77.6%) compared to non-malnourished children (51.8%) (**p* *= 0.0003). Prolonged hospital stay (aOR=1.12, 95% CI = 1.05–1.19, *p* = 0.001), malnourishment (aOR=3.15, 95% CI = 1.61–6.19, *p* = 0.001), and a history of antibiotic use (aOR=2.28, 95% CI = 1.15–4.54, *p* = 0.018) were independently associated with the carriage of ESBL-PE. Furthermore, we observed that ESBL-producing *E. coli* isolated from malnourished children had high resistance rates of 75% and 65% against aztreonam and sulfamethoxazole/trimethoprim, respectively, while ESBL-producing *K. pneumoniae* isolated from the same group exhibited high resistance rates of 84.6% and 69.2%.

**Conclusion:**

The present study revealed that children under five years old with diarrhea have a higher rate of ESBL-PE fecal carriage. Furthermore, prolonged hospital stays, malnutrition, and a history of antibiotic use are independently associated with ESBL-PE carriage. We also report high resistance rates of ESBL-PE isolated from malnourished children. These findings emphasize the importance of enhancing infection prevention and control measures to mitigate the spread of multidrug-resistant pathogens in both hospital and community settings.

## Introduction

The group of beta-lactamase-producing bacteria is primarily recognized for its ability to produce extended-spectrum beta-lactamase enzymes, which resist antibiotics such as penicillin, cephalosporins, and monobactam [1]. The presence and infections caused by extended-spectrum beta-lactamase-producing Enterobacteriales (ESBL-PE) remain a global threat, steadily increasing and linked to high rates of morbidity and mortality [2].

The global prevalence of ESBL-PE has raised significant concern, with a systematic review revealing a pooled prevalence of 25.4%. This high prevalence is primarily associated with the increased irrational use of antibiotics [1,3,4]. The magnitude of ESBL-PE varies by geographical location; the prevalence of ESBL-PE carriage in the community ranges from 20% to 70% in Asia and from 10% to 15% in South America [1,5].

Africa is one of the continents with a high carriage rate, with an estimated 110 million people being ESBL-PE carriers [5,6]. A systematic review found an overall pooled prevalence of 28%, with East Africa reporting even higher rates (29%) [5]. In a previous study conducted in Tanzania, the ESBL-PE carriage rate among hospitalized children under five years old with fever was 56% [7]. Studies in other regions of Africa, such as Nigeria, have also revealed an alarming ESBL-PE carriage rate of 37.5% in children under five, highlighting the role of prior antibiotic use and non-prescription antibiotic usage as key risk factors for colonization [8]. In Africa, the high carriage rate is attributed to factors such as high population density, poor access to drinking water, and poverty.

Evidence shows that children under five years old, particularly those who are malnourished, face a higher risk of infectious diseases, including wound infections, urinary tract infections, gastroenteritis, meningitis, pneumonia, and septicemia [9,10]. This risk arises from a compromised immunological status, which makes them more susceptible to bacterial infections, leading to increased antibiotic use among these children [11–14]. Antibiotics are recommended as part of the systemic treatment for children with severe acute malnutrition (SAM) [15,16]. However, the use of antibiotics in SAM remains contentious due to the risk of fostering carriage and the potential spread of multidrug-resistant bacteria [17]. Moreover, children carrying ESBL-PE contribute to its transmission due to poor personal hygiene, thereby spreading pathogens to healthcare providers and the community [18–20].

In our settings, there is limited information on the extent of ESBL-PE among children under five years old, including those who are malnourished. Therefore, this study aimed to determine the fecal carriage of ESBL-PE and the associated factors among children under five with diarrhea in a tertiary hospital in Tanzania. Understanding the fecal carriage of ESBL-PE and its associated factors is beneficial for healthcare providers in the clinical care and management of children under the age of five. Additionally, the findings from the assessment of ESBL-PE are crucial for understanding the epidemiology and potential carriage burden. Such information assists in designing and emphasizing the need to implement hospital prevention and infection control strategies to avert further occurrences and the spread of ESBL-PE and other resistant bacteria.

## Materials and methods

### Study design and settings

The study was a hospital-based cross-sectional study conducted at Muhimbili National Hospital (MNH) in Dar es Salaam, Tanzania, from March 1, 2022, to May 31, 2022. MNH is the largest tertiary hospital in Tanzania, with a capacity of 1,500 beds. Additionally, the hospital serves as a research and teaching institution, attending to 1,000–1,200 outpatients daily and admitting 1,000–1,200 patients weekly. The study took place in a ward designated for admitting children with diarrhea, both malnourished and non-malnourished, which has a capacity of 16 beds. The average number of patient admissions in the diarrhea ward per month was approximately 40 patients.

### Study population

The study focused on hospitalized children under the age of five who presented with diarrhea. This group was considered the study population because many children of this age cannot maintain personal hygiene independently. As a result, they can carry ESBL-PE and may transmit it to healthcare providers and the community. We obtained written informed consent from the parents and guardians of all children under the age of five with diarrhea.

### Sample size and sampling technique

The sample size was calculated using the Kish and Leslie formula (1965): n = z^²^p (1-p)/e², where n represents the sample size, z is the standard normal deviate corresponding to a 95% confidence interval (z value = 1.96), e is the margin of error at 6%, and p is the proportion of ESBL-PE (17.1%) in children from a previous study in Addis Ababa, Ethiopia [21]. Thus, the estimated minimum sample size was 151 participants. However, considering the assumption of an 83% response rate, the sample size was estimated to be 181 (151/0.83) under-five children with diarrhea. A convenient sampling technique was employed to recruit the available participants for this study, whereby newly admitted children under five years of age who met the inclusion criteria were enrolled until the required sample size was achieved.

### Data collection

We utilized a structured questionnaire to gather sociodemographic data (sex, place of residence, and parent or guardian education level) and documented risk factors for ESBL-PE carriage (HIV status, nutritional status, and history of antibiotic use). The child’s clinic card provided information such as age, current body weight, height, and immunization status. Malnourished children aged six months and older were identified using mid-upper arm circumference (MUAC), while those under six months were assessed using the age-for-weight Z-score (WAZ) [22,23].

### Specimen collection

The stool containers, which held a plastic spatula (Kishan Industries Ltd, Tanzania, Batch No. 001/2022), were used for specimen collection. After labeling a stool container with the participant’s identification number and date, parents or guardians were instructed to collect stool samples while avoiding contamination with urine. Subsequently, stool samples were transported to the Microbiology Laboratory at Muhimbili University of Health and Allied Sciences within one hour for processing, using a cool box.

### Laboratory procedures

#### ESBL-PE isolation.

ESBL-PE were isolated from stool samples by inoculating them onto MacConkey agar (MCA) plates supplemented with 2.0 µg/ml ceftazidime and incubated aerobically at 37 ºC for 24 hours. The presence of ceftazidime in MCA allowed for the growth of ESBL-PE while inhibiting the growth of non-ESBL-PE.

#### Phenotypic ESBL-PE confirmation.

The isolates from stool were confirmed as ESBL-PE using the Double Disc Synergy Test [24]. Briefly, a suspension of microorganisms was prepared and adjusted to match the 0.5 McFarland standard. A lawn culture was established on Mueller-Hinton agar (MHA). Ceftazidime (30 μg) and cefotaxime (30 μg) discs were positioned 20 mm apart from an amoxicillin-clavulanic acid (30 μg) disc. The plates were incubated aerobically at 37 ºC for 16–18 hours. Isolates were confirmed as ESBL-PE when the zone of inhibition around any of these third-generation cephalosporin discs displayed a distinct increase toward the amoxicillin-clavulanic acid disc.

#### Isolate identification.

The preliminary identification of bacterial isolates was based on colonial morphology and Gram staining. Subsequently, conventional biochemical tests were employed to identify colonies that were subcultured on nutrient agar. The following biochemical tests were used for identification: oxidase test, carbohydrate utilization tests, indole production test, urease test, citrate utilization test, Kligler iron agar (KIA) test, H_2_S production test, and motility test.

#### Antimicrobial susceptibility test.

All isolated bacteria underwent antimicrobial susceptibility testing with four antibiotics: aztreonam (30 μg), gentamicin (10 μg), sulfamethoxazole-trimethoprim (1.25/23.75 μg), and meropenem (10 μg). The zone of inhibition was interpreted according to the Clinical and Laboratory Standards Institute (CLSI) guidelines from 2021 [25]. The Kirby-Bauer disc diffusion method was employed, with suspensions of microorganisms prepared to match the 0.5 McFarland standard. A lawn culture was prepared on Mueller-Hinton agar (MHA), and antibiotic discs to be tested were placed equidistantly from each other and incubated overnight at 37 ºC. The diameters of the inhibition zones and their interpretations for each antibiotic tested were used to calculate the resistance pattern for each isolate [25].

#### Quality control.

Stool samples were collected following standard procedures to prevent contamination with non-target bacteria. Stains and reagents were clearly labeled, dated, and properly stored. The operating temperatures of the refrigerator and incubator were monitored and documented. The culture media were prepared according to the manufacturer’s instructions and tested for performance and sterility before use. To standardize the turbidity of the bacterial suspension for ESBL-PE tests, a 0.5 McFarland standard was used as per the 2021 CLSI guideline [25]. The *Klebsiella pneumonia* (ATCC 700603) and *Escherichia coli* (ATCC 25922) served as ESBL-positive and negative control bacterial strains, respectively.

### Data management and analysis

Participants’ sociodemographic and clinical information, along with laboratory results, were recorded in Microsoft Excel software version 2010 for editing and coding before being exported to STATA version 15.1 for analysis. The overall fecal carriage of ESBL-PE was calculated by dividing the number of children identified as having ESBL-PE by the total number of children recruited for the study. Additionally, the fecal carriage of ESBL-PE among subgroups of diarrheal malnourished and non-malnourished children was calculated by dividing the number of children with ESBL-PE in each subgroup by the total number of children recruited in each subgroup.

Descriptive analysis employed frequency and proportion for categorical variables, alongside the median and interquartile range (IQR) for continuous variables. A chi-square test was conducted to determine the statistical differences in ESBL-PE carriage across categorical variables, while the Mann–Whitney U test was used for continuous variables. Variables with a p-value < 0.20 in bivariable analysis, those identified as risk factors for ESBL-PE carriage, and suspected confounders were incorporated into a multivariable logistic regression model. This multivariable logistic regression analysis identified predictors independently associated with ESBL-PE carriage among children under five with diarrhea. A *p-value* <0.05 was set as the threshold for statistical significance in all analyses.

### Ethical consideration

Ethical clearance was obtained from the Senate Research and Publications Committee of the Muhimbili University of Health and Allied Sciences (MUHAS) (MUHAS-REC-02-2022-967). Permission to conduct the study was granted by the MNH administration. Written informed consent was acquired from the parents or guardians of each participant prior to their enrolment in the study. The confidentiality of the study participants was maintained by using codes instead of participants’ names.

## Results

### Social-demographic and clinical information of study participants

The study enrolled 181 children under the age of five with diarrhea. The participants’ median age was 29 months (IQR: 20–37), and they had a male-to-female ratio of 1:1. Approximately 50% of parents or guardians had a low level of education, limited to primary school. Furthermore, the median number of hospital-stay days before enrollment was 5 days (IQR: 2–12). Additionally, 39.8% (72/181) of participants were HIV positive, and 54.4% (98/181) were malnourished. Moreover, 49.5% of participants had used antibiotics within the 4 weeks prior to enrollment in the study (Table 1).

**Table 1:** Social demographic and clinical information of study participants (N = 181).

Variable		N	Percent (%)
**Age(months)**	Median (IQR)	29 (20-37)	
**Sex**	Male	98	54.1
	Female	83	45.9
**Parent education level**	Primary School	98	54.1
	Secondary School	51	28.2
	College/University	32	17.7
**Duration of hospitalization (days)**	Median (IQR)	5 (2-12)	
**HIV status**	Positive	72	39.8
	Negative	109	60.2
**Nutrition status**	Non-malnourished	83	45.6
	Malnourished	98	54.4
**Antibiotic use (past 4 weeks)**	Yes	90	49.7
	No	91	50.3

### ESBL-PE carriage among under-5-year-old diarrheic children

Of the 181 children’s stool samples processed for culture, 119 (65.7%) showed bacterial growth on the initial screening media for ESBL-PE. These 119 samples yielded 125 suspected ESBL-PE isolates. Among these 125 isolates, 120 (96.0%) were confirmed as ESBL-PE, with two confirmed ESBL-PE isolates originating from a single sample; therefore, 119 out of 181 (65.7%) participants had ESBL-PE fecal carriage. The predominant ESBL-PE isolate was *E. coli,* accounting for 89/120 (74.2%), followed by *K. pneumoniae* at 21/120 (17.5%), *K. oxytoca* at 8/120 (6.7%), and Citrobacter spp at 2/120 (1.7%).

The carriage of ESBL-PE among malnourished children was significantly higher at 76.7% (76/98) compared to non-malnourished children at 51.8% (43/83) (*p* < 0.001). The antimicrobial susceptibility pattern of ESBL-PE isolated from participants was also assessed. It was observed that ESBL-producing *E. coli* from malnourished children exhibited a high resistance rate against aztreonam (75.0%) and sulfamethoxazole/trimethoprim (65.0%). Similarly, ESBL-producing *K. pneumoniae* from the same group demonstrated high resistance rates against aztreonam (84.6%) and sulfamethoxazole/trimethoprim (69.2%).

In the present study, we observed a significant difference in the median duration of hospitalization between children with ESBL-PE fecal carriage (median: 7, IQR: 2–13 days) and those without ESBL-PE fecal carriage (median: 3, IQR: 1–6 days) (*p* = 0.0007). Furthermore, there was a significant difference in ESBL-PE carriage among children with a history of antibiotic use in the past four weeks, 72.5% (66/91), compared to those without that history, 58.9% (53/90) (*p* = 0.032) (Table 2).

**Table 2:** Fecal carriage of ESBL-PE by children’s and parents’ socio-demographic and clinical factors.

Variable	Total number (N)	Fecal ESBL-PE carriage		95% CI	*p*-value
		Yes, n (%)	No, n (%)		
**Median age in months (IQR)**	29 (20-37)	28 (20-37)	29 (34.9)	**NA**	0.6526
**Sex**					
Male	98	65 (66.3)	33 (33.7)	0.56-0.76	0.876
Female	83	54 (65.1)	29 (34.9)	0.54-0.75	
**Parent’s education level**					
Primary	98	69 (70.4)	29 (29.6)	0.60-0.79	0.783
Secondary	51	29 (56.9)	22 (43.1)	0.42-0.71	
College/University	32	21 (65.6)	11 (34.4)	0.47-0.81	
**Median duration of hospitalization (days)**	5 (2-12)	7 (2-13)	3 (1-6)	NA	**<0.001**
**Nutrition status**					
Non-malnourished	83	43 (51.8)	40 (48.2)	0.41-0.63	**<0.001**
Malnourished	99	76 (76.7)	23 (23.2)	0.67-0.85	
**HIV status**					
Positive	72	47 (65.3)	25 (34.7)	0.53-0.75	1.000
Negative	109	72 (66.1)	37 (33.9)	0.56-0.75	
**History of Antibiotic use (past 4 weeks)**					
No	90	53 (58.9)	37 (41.1)	0.48-0.69	**0.032**
Yes	91	66 (72.5)	25 (27.5)	0.62-0.81	
**Overall**	181	119 (65.7)	62 (34.3)	0.58-0.73	

*p-value (Chi-square test).*

### Factors associated with fecal carriage of ESBL-PE

Bivariable and multivariable logistic regression analyses were conducted to explore the factors associated with ESBL-PE fecal carriage among diarrheic children at MNH. The logistic regression analysis revealed that for each additional day of hospitalization, the odds of having ESBL-PE carriage among diarrheic children increased by 1.12 (aOR = 1.12, 95% CI = 1.05–1.19, *p* = 0.001). Additionally, children with a history of antibiotic use in the past four weeks had double the risk of ESBL-PE fecal carriage compared to those without such a history (aOR = 2.28, 95% CI = 1.15–4.54, *p* = 0.018). Furthermore, malnourished children faced three times the risk of ESBL-PE carriage compared to their non-malnourished counterparts (aOR=3.15, 95% CI = 1.61–6.19, *p* = 0.001) (Table 3).

**Table 3:** Bivariable and Multivariable logistic regression for the factors associated with ESBL-PE fecal carriage.

Variable	ESBL-PE fecal carriage n (%)	Bivariable logistic regression		Multivariable logistic regression	
		cOR(95%CI)	*p*-value	aOR(95%CI)	*p*-value
**Duration of hospitalization (days)**	119	1.11 (1.04-1.17)	0.001	1.12 (1.05-1.19)	0.001
**Nutrition status**					
Non-malnourished	43(51.8)	Ref		Ref	
Malnourished	76(77.6)	3.21(1.69-6.10)	<0.001	3.15(1.61-6.19)	0.001
**History of Antibiotic use (past 4 weeks)**					
No	53(58.2)	Ref		Ref	
Yes	66(73.3)	1.97(1.05-3.69)	0.034	2.28(1.15-4.54)	0.018

## Discussion

In several developing countries, including Sub-Saharan Africa, the prevalence of ESBL-PE fecal carriage among children under five years old is high, with an elevated risk among malnourished children. Our study found that approximately two-thirds of children under five years of age with diarrhea carried ESBL-PE. These findings contrast with studies conducted in Tanzania among children hospitalized for diarrhea, which reported a lower prevalence of 34%, as well as another study in Addis Ababa, Ethiopia, that indicated a 17.1% prevalence [21]. The prevalence is quite similar to that found in a study by Kibwana *et al.,* who reported a 56% ESBL-PE carriage rate among children under five years of age hospitalized with fever [7]. The high prevalence of ESBL-PE fecal carriage in this study may be attributed to the significant antibiotic exposure experienced by most admitted children, which could have facilitated the establishment of resistance genes associated with ESBL [19,24,26]. This further suggests an increased risk of ESBL-PE acquisition with prolonged hospital exposure, potentially contributing to the high rate of ESBL-PE fecal carriage reported in this study [24]. Nonetheless, the elevated ESBL-PE carriage prevalence identified here reflects the growing threat of multidrug-resistant bacteria in resource-limited settings [27]. This situation poses a significant challenge for managing childhood diarrheal diseases, which are often treated with empirical antibiotics that may be ineffective against ESBL-PE.

Further stratification analysis in our study revealed that the magnitude of ESBL-PE carriage in malnourished children was significantly higher than that in non-malnourished children. A similar finding was reported in a study conducted in Kenya by Njoroge et al., which showed a high prevalence of ESBL-PE carriage among malnourished children (39%) compared to non-malnourished children (7%) [20]. Also, in Senegal, Ndir *et al*. reported findings similar to those of the current study, where malnourished children were found to be at risk of acquiring ESBL-PE [28]. These differences in magnitude between the two subpopulations may stem from impaired immunity, making malnourished children more susceptible to bacterial infections and more prone to exposure to antibiotics that promote the acquisition and spread of multidrug-resistant bacteria. Additionally, as reported by Holowka *et al*., the weakening of intestinal barrier function and adaptive and innate immunity in malnourished children increases the threat of infection with intestinal-derived pathogens, such as ESBL-PE. Furthermore, the need to treat infections exposes bacteria to frequent antibiotic use, resulting in the development of resistant mechanisms that prevent the activity of these drugs. Moreover, the frequent hospitalization of malnourished children results in nosocomial transmission of ESBL-PE among themselves [29].

This study found that among children under the age of five, *E. coli* was the most frequently isolated ESBL-PE, followed by *K. pneumoniae* (21/120, 17.5%), *K. oxytoca (8/120, 6.7%),* and *Citrobacter spp.* (2/120, 1.7%). This finding aligns with reports from studies conducted in similar settings in Kenya and Tanzania [20,30–32]. The predominance of *E. coli* isolates among ESBL producers has also been noted by Onduru *et al*. in a meta-analysis review spanning Eastern, Central, and Southern African countries [33]. The study by Ndir *et al.* from Senegal indicated that *Enterobacter spp.* were the predominant isolates at 88%, followed *by Klebsiella* spp at 82%, and E. coli at 58.3% [28]. The similarities and differences observed in the predominance of ESBL-PE isolates might be attributed to variations in the geographical locations of the studies conducted.

In determining the factors associated with ESBL-PE fecal carriage among diarrheic children, it was observed that the duration of hospitalization, malnutrition status, and history of antibiotic use were independently linked to ESBL-PE fecal carriage in children under five years old. These findings are comparable to those reported in a study conducted in Lebanon, which indicated that prolonged hospitalization was associated with ESBL-PE fecal carriage [34]. Additionally, similar studies conducted in Kenya and elsewhere have reported that the risk of ESBL-PE carriage increases with a history of antibiotic use and poor nutritional status [20,30,31,34–36]. The connection between extended hospitalization and ESBL-PE carriage likely reflects exposure to contaminated environments and increased healthcare interventions, including antibiotic administrations [31,37]. Moreover, the relationship between a history of antibiotic use and ESBL-PE carriage confirms existing knowledge regarding the role of antibiotic exposure in developing resistance [38]. Furthermore, the association between nutritional status and susceptibility to antibiotic-resistant bacteria stems from the fact that poor nutritional status can compromise gut barrier function and weaken the immune system, potentially facilitating colonization by ESBL-PE [29].

This finding emphasizes the importance of prudent antibiotic use, highlighting the need for targeted therapies and avoiding unnecessary self-prescriptions, especially in the pediatric population, to minimize bacteria’s exposure to commonly used drugs that may lead to the development of ESBL-producing bacteria. Furthermore, the findings suggest a need to enhance infection prevention and control measures to reduce the spread of multidrug-resistant pathogens in both hospital and community settings. This can be achieved by focusing on hand hygiene, thorough cleaning of surfaces, proper wound care, and safe handling of contaminated items, as well as avoiding the sharing of personal items.

The present study has several limitations. First, we were unable to conduct molecular testing to identify the resistance genes due to limited funding. However, we have stored the ESL-PE isolates under appropriate and standard conditions for future molecular characterization testing. Additionally, this study did not determine the source of ESBL-PE acquisition, whether from the community or the hospital environment. Future studies should aim to identify the specific ESBL-PE genotypes circulating at MNH and explore the potential of nutritional interventions to improve gut health and reduce susceptibility to ESBL-PE colonization in malnourished children.

## Conclusion

The fecal carriage of ESBL-PE among diarrheic children at MNH is high, with factors such as antibiotic use, nutritional status, and prolonged hospitalization linked to this condition in this group. Furthermore, *E. coli, K. pneumoniae, K. oxytoca, and Citrobacter spp.* were identified as the isolated ESBL-PE. These findings have significant implications for public health interventions in Tanzania. Addressing ESBL-PE carriage in diarrheic children should involve a multifaceted approach, including enhanced infection prevention in healthcare settings, targeted nutritional interventions, and antibiotic stewardship programs. Additionally, the findings suggest prioritizing children for ESBL screening to prevent ESBL-PE-associated infections, reduce transmission to healthcare workers, and mitigate potential spread in hospital settings and the community.
